# Cocultivation of White-Rot Fungi and Microalgae in the Presence of Nanocellulose

**DOI:** 10.1128/spectrum.03041-22

**Published:** 2022-09-26

**Authors:** Carolina Reyes, Zsófia Sajó, Miriam Susanna Lucas, Ashutosh Sinha, Francis W. M. R. Schwarze, Javier Ribera, Gustav Nyström

**Affiliations:** a Laboratory for Cellulose and Wood Materials, Empa, Dübendorf, Switzerland; b Scientific Center for Light and Electron Microscopy (ScopeM), ETH Zurich, Zürich, Switzerland; c Laboratory for Cellulose and Wood Materials, Empa, St. Gallen, Switzerland; d Department of Health Science and Technolgy, ETH Zürich, Zürich, Switzerland; Broad Institute

**Keywords:** *Chlorella vulgaris*, *Scenedesmus vacuolatus*, TEMPO, cellulose, nanofibril, 3D-printing, algae, white-rot fungi

## Abstract

Cocultivation of fungi and algae can result in a mutualistic or antagonistic interaction depending on the species involved and the cultivation conditions. In this study, we investigated the growth behavior and enzymatic activity of two filamentous white-rot fungi (Trametes versicolor and Trametes pubescens) and two freshwater algae (Chlorella vulgaris and Scenedesmus vacuolatus) cocultured in the presence of TEMPO (2,2,6,6-tetramethylpiperidine-1-oxyl radical) oxidized cellulose nanofibrils (CNF) and cellulose nanocrystals (CNC). The growth of fungi and algae was studied in liquid, agar medium, and 3D-printed nanocellulose hydrogels. The results showed that cocultures grew faster under nutrient-rich conditions than in nutrient-depleted conditions. Key cellulose-degrading enzymes, including endoglucanase and laccase activities, were higher in liquid cocultures of T. versicolor and S. vacuolatus in the presence of cellulose compared to single cultures of fungi or algae. Although similar results were observed for cocultures of T. pubescens and C. vulgaris, laccase production diminished over time in these cultures. Fungi and algae were capable of growth in 3D-printed cellulose hydrogels. These results showed that cellulase enzyme production could be enhanced by cocultivating white-rot fungi with freshwater algae under nutrient-rich conditions with TEMPO-CNF and CNC. Additionally, the growth of white-rot fungi and freshwater algae in printed cellulose hydrogels demonstrates the potential use of fungi and algae in hydrogel systems for biotechnological applications, including biofuel production and bio-based fuel cell components.

**IMPORTANCE** Depending on the conditions used to grow fungi and algae in the lab, they can interact in a mutually beneficial or negative way. These interactions could stimulate the organisms to produce enzymes in response to the interaction. We studied how wood decay fungi and freshwater algae grew in the presence and absence of cellulose, one of the basic building blocks of wood. How fungi and algae grew in 3D-printed cellulose hydrogels was also tested. Our results showed that fungi and algae partners produced significantly larger amounts of enzymes that degraded cellulose when grown with cellulose than when grown alone. In addition, fungi and algae were shown to grow in dense nanocellulose hydrogels and could survive the shear conditions during gel structuring while 3D-printing. These cultures could potentially be applied in the biotech industry for applications like energy production from cellulose, biofuel production, and bioremediation of cellulose material.

## INTRODUCTION

In nature, mutualistic interactions are well documented for Ascomycete fungi (e.g., lichens), which are believed to have formed a symbiosis with algae over millions of years (1, 2). Algae, including Chlorella, diatoms, and kelp, are comprised of unicellular and multicellular organisms and are found in both aquatic and terrestrial ecosystems. Diverse members of wood-decaying Basidiomycetes have been found to harbor different species of algae on their basidiocarps, the spore-forming fruiting body of a mushroom (3, 4). Mutualistic interactions between fungi and algae could lead to an exchange of nitrogen and oxygen, produced by the algae, with sugars, minerals, and CO_2_, produced by the fungi as a product of respiration or fermentation (5). Wood in general contains low levels of nitrogen compared to carbon (N:C ratio, 1:350 to 1:1250) (6). Thus, having an algal partner could be advantageous to wood-inhabiting fungi.

Prior laboratory studies showed that yeast and filamentous fungi (Ascomycetes) can form a mutualistic symbiosis with algae in closed cultivation systems to various degrees (7, 8). Excluding certain nutrients from the coculture growth medium, forced certain fungi and algae to rely on each other for carbon and nitrogen and promoted a symbiotic association. This association could be transient and could change depending on nutrient availability, pH, or temperature. In another study, the cultivation of the alga Nannochloropsis oceanica with the fungus Mortierella elongata prompted the internalization of the algae cells into the fungal mycelium (9). However, sometimes algal and fungal interactions have led to antagonistic growth (8). In the study of Klawonn et al. (10), microparasitic fungi associated with diatoms derived one hundred percent of their carbon from their diatom host. Cocultures of fungi and algae can also result in increased enzymatic activity compared to single cultures. Cocultivation studies between microalgae and fungi have also shown that their interactions can lead to increased production of high-value compounds, normally not produced in single cultures, with application in pharmaceutical, cosmetic, and brewery industries (11). Until now, studies focusing on the cocultivation of white-rot fungi and algae have mainly focused on their use in wastewater removal (12–14).

Previously, we showed that TEMPO (2,2,6,6-tetramethylpiperidine-1-oxyl) oxidized cellulose nanofibrils (CNF) and cellulose nanocrystals (CNC) did not inhibit the activity of lignocellulose enzymes in cultures of the white-rot fungi studied here (15). TEMPO-oxidized CNF and CNC materials are plant-derived nanomaterials that are commercially available to make biodegradable, biorenewable, biocompatible, and bio-based materials (16). Compared to other bio-based materials, cellulose is highly abundant, inexpensive, a tunable chemical, and has good mechanical and physical properties (17). In recent years, the production cost of TEMPO-oxidized CNF materials has decreased, making them more attractive for biotechnological applications (18).

Although it is known that exposure of selected white-rot fungi to nanocellulose does not inhibit enzyme activity, similar information about fungal-algal cocultures in nanocellulose matrix/gels is lacking. The use of cellulose-based hydrogels to cocultivate fungi and algae grants the possibility to 3D-print these two cell types in a porous matrix, allowing to study direct cell interactions between algae and fungi in the matrix. Until recently, 3D-printing technology has been applied to studies of bacteria (19), algae (20), yeast (21), and white-rot (22) as single cultures or they have been studied together in a synthetic hydrogel system (23, 24).

In this study, different species of white-rot fungi and different types of freshwater algae were cocultivated in a liquid medium and 3D-printed hydrogels. Their interaction was assessed in the presence and absence of TEMPO-oxidized CNF and CNC by light and confocal laser scanning microscopy (CLSM) and enzyme activity assays. TEMPO-oxidized CNF and CNC material can potentially serve as a nutrient source in manufacturing fungal-algal bio-based hydrogels for energy production from cellulose and bioremediation of cellulose material. However, its successful application relies on its biocompatibility. Thus, our study is important for demonstrating the biocompatibility of TEMPO-oxidized CNF and CNC with fungal-algal coculture systems.

## RESULTS

### Light microscopy images of cocultures after 21 days of cultivation.

**FIG 1:**
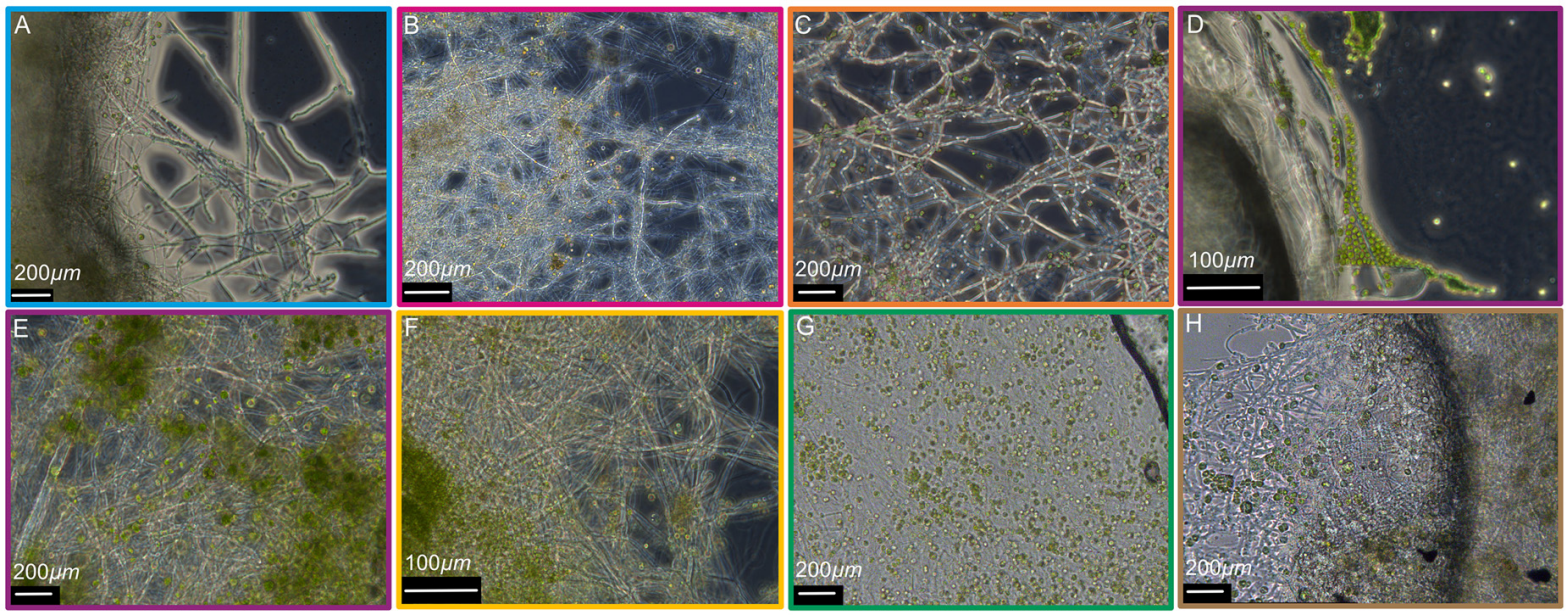
Microscopic image showing various combinations of white-rot fungi and freshwater algae that were grown in 1:1 SK:ME medium mixture after 21 days of cocultivation. (A, blue) T. pubescens 220 and C. vulgaris. (B, magenta) T. pubescens 220 and S. vacuolatus. (C, orange) T. versicolor 159 and S. vacuolatus. (D and E, purple) R. vitreus 643 and C. reinherdetti. (F, yellow) R. vitreus 643 and C. vulgaris. (G, green) R. vitreus 643 and S. vacuolatus. (H, brown) G. adspersum 003 and S. vacuolatus. (A to C, E, and G) Imaged using a Leica HC PL FLUOTAR 20×/0.50 DRY lens objective. (D and F) Imaged using a Leica HC PL FLUOTAR 10×/0.30 DRY lens objective.

The coculture experiments were initiated by combining different white-rot fungi and freshwater algae in long-term closed culture incubations and after some time, visualizing them using light microscopy. After 21 days of cocultivation, algae were observed as clusters and individual cells on top of fungal filaments. The alga Chlorella vulgaris appeared as individual cells and in association with the filaments of the white-rot fungus Trametes pubescens 220 (Fig. 1A). The alga Scenedesmus vacuolatus appeared to be embedded in T. pubescens 220 filaments (Fig. 1B) and in the filaments of the white-rot fungus Trametes versicolor 159 (Fig. 1C). Similarly, cells of the white-rot fungus Rigidoporus vitreus 643 formed clusters on top of the cells of the algae Chlamydomonas reinhardtti (Fig. 1D and E), C. vulgaris (Fig. 1F), and S. vacuolatus (Fig. 1G). The white-rot fungus Ganoderma adspersum 003 also formed clusters on top of S. vacuolatus cells (Fig. 1H).

### Confocal microscopy images of select cocultures.

To determine the viability of fungi and algae in cocultures with each other, we used a live dead stain, called SYTOX green, with confocal microscopy. SYTOX green permeates dead eukaryotic cells. Several cocultures were randomly selected from the above pairings for imaging. C. vulgaris appeared to attach to living T. pubescens 220 and R. vitreus 643 mycelial filaments (Fig. S1A and B in Supplemental File 1). S. vacuolatus appeared attached to living cells of T. versicolor 159 (Fig. S1C in Supplemental File 1). Similarly, S. vacuolatus appeared as single cells attached to living cells of G. adspersum 003 (Fig. S1D in Supplemental File 1) and in clusters with dead cells of R. vitreus 643 (Fig. S1E in Supplemental File 1).

### Growth of cocultures in liquid and solid mediums.

**FIG 2:**
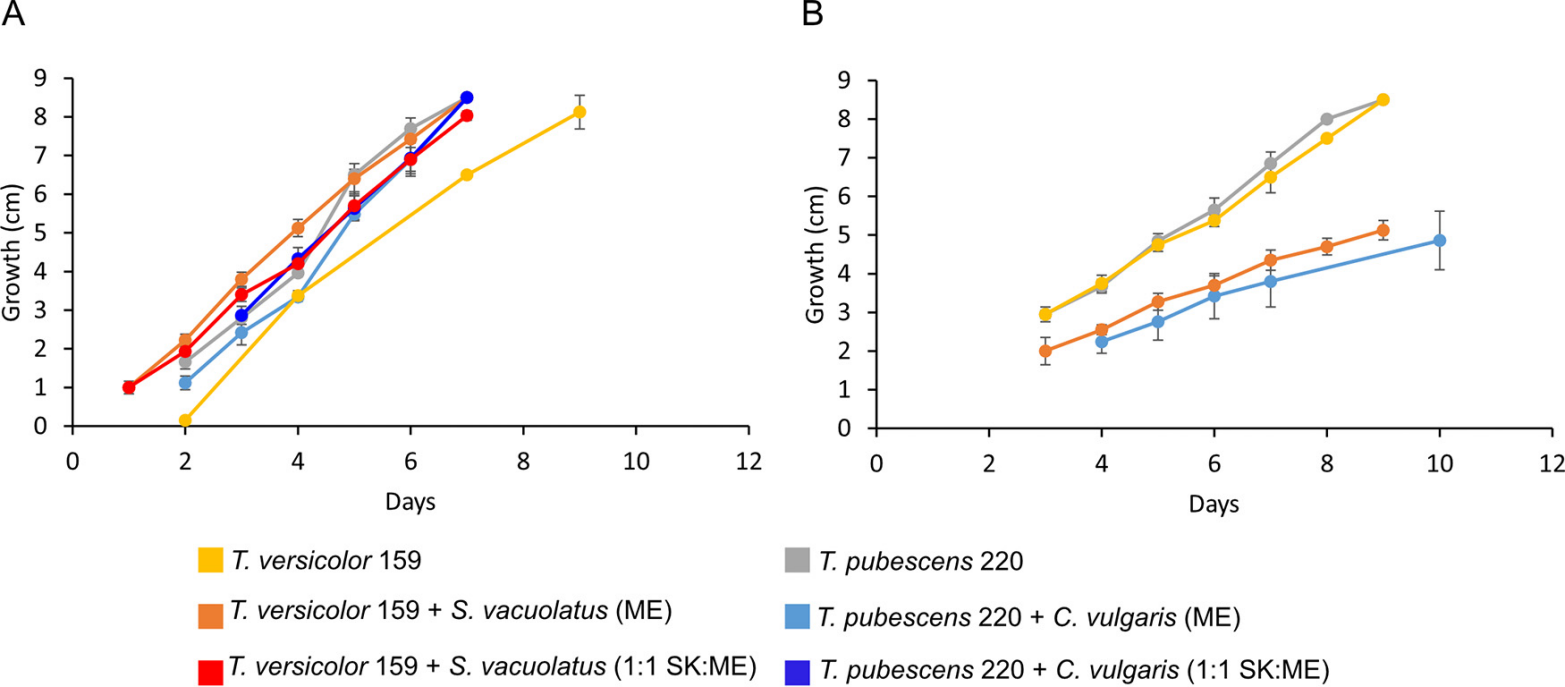
Growth rate of cocultures and fungal cultures in agar plates with or without nutrients. (A) Cocultures and fungal cultures in agar plates with 2% malt extract medium (ME) and 1:1 Sueoka and malt extract medium (1:1 SK:ME). (B) Cocultures and fungal cultures in agar plates with no additional nutrients.

We decided to continue investigating the fungi T. pubescens 220 and T. versicolor 159 as well as the algae C. vulgaris and S. vacuolatus because these fungi and algae appeared to grow quickly in 1:1 Sueoka-malt extract medium (1:1 SK:ME) and malt extract (ME) medium, facilitating growth experiments. In addition, species of *Trametes* are often found associated with freshwater algae in nature making them the most ideal samples to study (3, 4, 25). Of the fungal cocultures initially analyzed, T. pubescens 220, T. versicolor 159, and their algal partners C. vulgaris and S. vacuolatus grew rapidly in 1:1 SK:ME (1.3 cm/day and 1.4 cm/day, respectively) and ME conditions (1.7 cm/day and 1.3 cm/day) comparable to single cultures of T. versicolor 159 (1.6 cm/day) and T. pubescens 220 (1.3 cm/day) (Fig. 2A). However, under conditions with only water agar (no nutrients), T. pubescens 220, T. versicolor 159, and their algal partners C. vulgaris and S. vacuolatus (0.57 cm/day and 0.52 cm/day, respectively) grew slower compared to T. versicolor 159 (0.8 cm/day) and T. pubescens 220 (1 cm/day) (Fig. 2B).

### Transmission electron microscopy and scanning electron microscopy visualization of cocultures.

**FIG 3:**
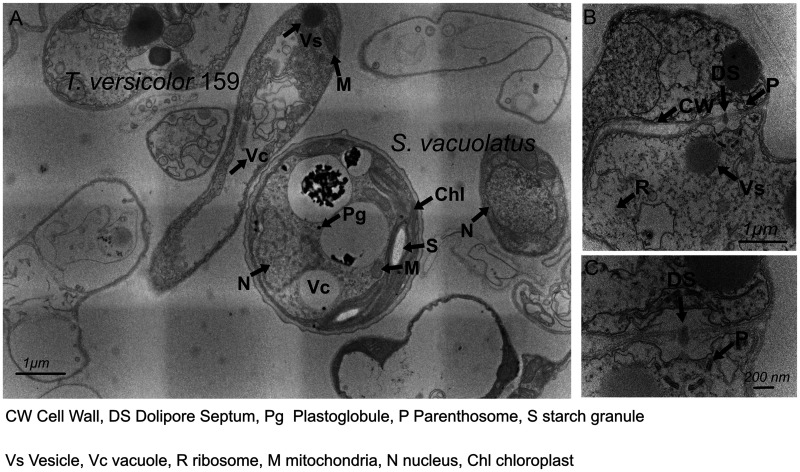
TEM images of T. versicolor 159 and S. vacuolatus coculture. (A) A cell of T. versicolor 159 lies next to a single cell of S. vacuolatus. A smaller cell of S. vacuolatus appears to the right. Empty fungal cells, attributed to cell death, appear in the surrounding area. (B) A close-up of a sample containing only T. versicolor 159 for comparison showed signs of cell division, in particular the dolipore septum. (C) A close-up of the cell in (B) showed the dolipore septum as a dashed circle.

Using transmission electron microscopy (TEM) imaging, cocultures could be further imaged at higher magnification and resolution to determine if algae had become internalized inside fungal cells in the process of cocultivation in 1:1 SK:ME. Imaging results showed S. vacuolatus adjacent to T. versicolor 159 but no images showed internalization (Fig. 3A). Although both T. versicolor 159 and S. vacuolatus cell structures appeared intact, in the coculture sample, there were empty fungal cells present that were absent in the T. versicolor 159 control sample (Fig. 3A). These are dead cells in different stages of decomposition. T. versicolor 159 appeared to be dividing due to the presence of the dolipore septum which is indicative of cell wall division (Fig. 3B and C).

In addition to TEM imaging, we could further image the biomass of T. versicolor 159 and S. vacuolatus coculture using scanning electron microscopy (SEM). The results showed that S. vacuolatus attached to fungal filaments of T. versicolor 159 and spread out throughout a filamentous network (Fig. S2A and B in Supplemental File 1). Similar to other species of *Scenedesmus* (26, 27), the cell surface of S. vacuolatus appeared knotted (Fig. S2C and D in Supplemental File 1).

### Biocompatibility of cocultures with nanocellulose inks.

**FIG 4:**
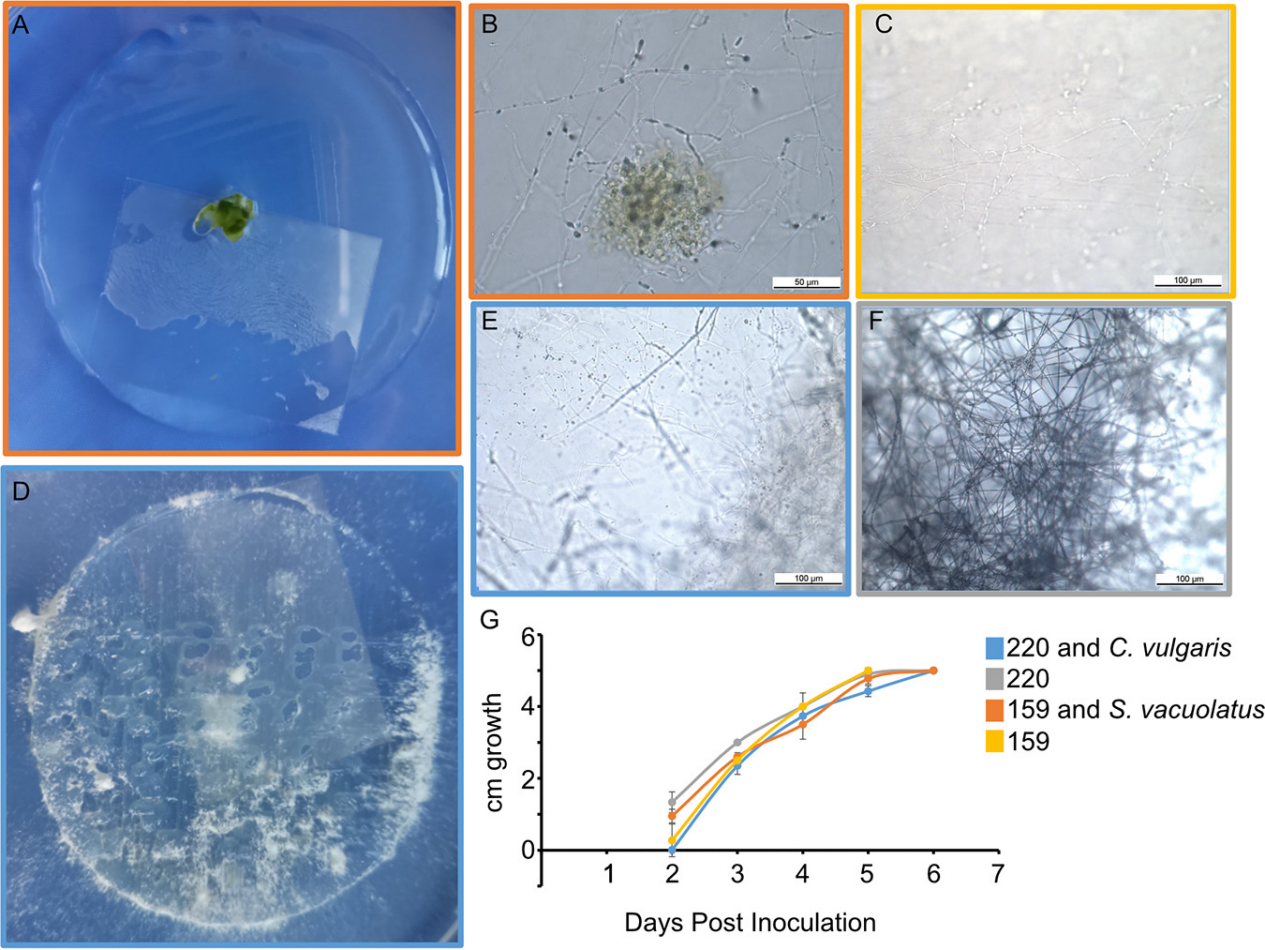
Growth of cocultures when grown on printed discs of TEMPO-oxidized CNF and CNC containing malt extract or mixed into the cellulose ink. A microscope coverslip was placed on top of the hydrogels for imaging. (A) A printed 10% wt hydrogel with T. versicolor 159 and S. vacuolatus biomass on top. (B) A 10% wt printed hydrogel of T. pubescens 220 and C. vulgaris showed overgrowth of T. pubescens 220 from within the printed hydrogel. (C) Light microscopy image showing the growth of S. vacuolatus as clusters and T. versicolor 159 filaments within the 10% wt printed hydrogels. (D) Light microscopic image of T. versicolor 159 alone growing within a 10% wt printed hydrogel for comparison. (E) Light microscopic image of C. vulgaris and filaments of T. pubescens 220 growing within the 10% wt hydrogels. (F) Light microscopic image of T. pubescens 220 alone growing within a 10% wt printed hydrogel for comparison. (G) Growth rates of T. versicolor 159 and S. vacuolatus and T. pubescens 220 and C. vulgaris on 5 cm diameter (1 mm thick) printed 10% wt hydrogels. The hydrogels were placed in Petri dishes with agar to keep them humid.

Our next goal was to determine if cocultures could grow in the presence of nanocellulose. We measured the growth rate of cocultures after seeding cells on top of printed inks (see Materials and Methods for preparation steps). Light microscopy revealed that S. vacuolatus cells were mostly concentrated in the center of the printed cellulose disc but appeared to remain attached to T. versicolor 159 mycelial filaments as the fungus spread out away from the center (Fig. 4A and B). Control cultures of T. versicolor159 alone showed similar mycelial filaments on the surface of the cellulose ink (Fig. 4C). C. vulgaris cells appeared entangled in T. pubescens 220 filaments but also appeared as single cells distributed throughout the cellulose ink (Fig. 4D and E). The control ink with T. pubescens 220 alone showed bundles of filaments but no evidence of single cell structures as in the coculture sample (Fig. 4F). Cocultures of the fungi T. versicolor 159, S. vacuolatus, T. pubescens 220, and C. vulgaris grew at the same rate as T. versicolor 159 and T. pubescens 220 alone in 1:1 SK:ME medium mixture containing cellulose (Fig. 4G).

To determine if algae and fungi could potentially be used in future 3D-printing applications involving nanocellulose, we tested their growth and viability after mixing C. vulgaris and T. pubescens 220 into inks and imaging them before and after printing. Results show that before printing, C. vulgaris appeared to be embedded within the cellulose ink (Fig. S3, row 1 in Supplemental File 1). T. pubescens 220 also appeared to be viable in the ink as no dead cells were observed (Fig. S3 row 2). After printing, C. vulgaris cells appeared to be less evident in the cell images and were not found as clusters as in the preprinted inks (Fig. S3, row 3 in Supplemental File 1). T. pubescens however, was found more often within printed inks as filaments and clusters (Fig. S3, row 4 in Supplemental File 1).

### Endoglucanase activity.

**FIG 5:**
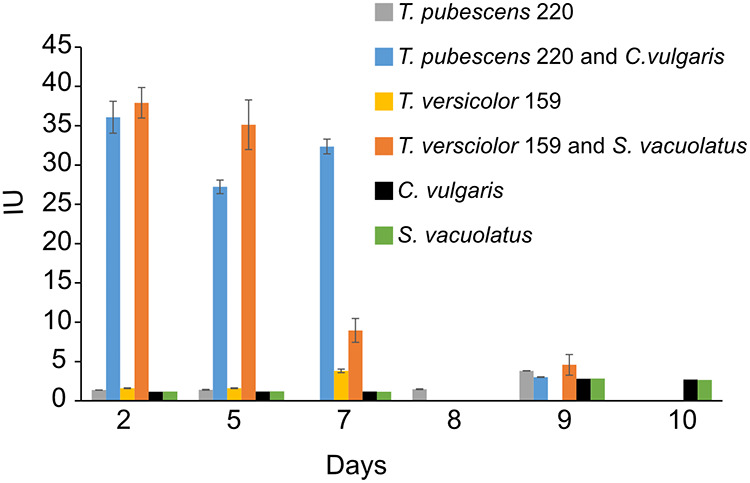
Endoglucanase activity on different days of the incubation period of cocultures, white-rot fungi, or algae when incubated with 1:1 SK:ME medium mixture and TEMPO-oxidized CNF (0.017% wt) and CNC (0.2% wt) under ambient conditions and red and blue artificial light. Cultures were initially shaken to stimulate growth but after 2 days they were left to stand for the duration of the experiment. Data represent an average of three biological replicates.

We were also interested in learning if cocultures could degrade cellulose inks because white-rot fungi can degrade lignocellulose material. To this end, we carried out a series of enzyme assays to determine if cocultures produced endoglucanase, a cellulase degrading enzyme. T. versicolor 159 and S. vacuolatus cocultures showed significantly higher endoglucanase activity (4.6 to 38 IU) compared to T. versicolor 159 control (1.6 to 3.8 IU). Enzyme activity was highest at the start of the experiment and decreased with time (Fig. 5). A similar trend was observed in the T. pubescens 220 and C. vulgaris cocultures (Fig. 5). Compared to the cocultures (3 to 36 IU), the fungi alone (1.4 to 3.8 IU) had lower endoglucanase activity. Algae alone showed the lowest endoglucanase activity (1.2 to 2.8 IU, C. vulgaris; 1.2 to 2.8 IU, S. vacuolatus) (Fig. 5).

### Laccase activity.

**FIG 6:**
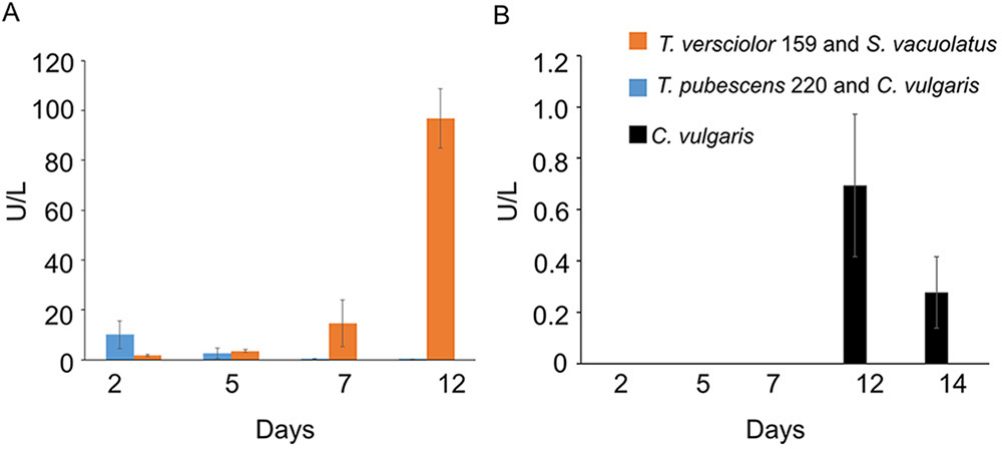
Laccase activity on different days of the incubation period of cocultures, white-rot fungi, or algae when incubated with 1:1 SK:ME medium mixture and TEMPO-oxidized CNF (0.017% wt) and CNC (0.2% wt) under ambient conditions and red and blue artificial light. (A) Laccase activity in cultures of T. versicolor 159 and S. vacuolatus as well as T. pubescens 220 and C. vulgaris. (B) Laccase activity in cultures of C. vulgaris. Cultures were initially shaken to stimulate growth but after 2 days they were left to stand for the duration of the experiment. Data represent an average of three biological replicates. Cultures were initially shaken to stimulate growth but after 2 days they were left to stand for the duration of the experiment. Data represent an average of three biological replicates.

Another enzyme that is often secreted by white-rot fungi during lignocellulose degradation is laccase. Thus, laccase activity was also assessed in the same cocultures as endoglucanase. Laccase activity was highest in cocultures compared to cultures of only fungi or algae (Fig. 6A and B). In T. pubescens 220 and C. vulgaris cocultures, laccase activity decreased with time (day 2, 10 U/liter; day 12, 0.2 U/liter). Contrasting results were observed in the T. versicolor 159 and S. vacuolatus incubations. In these incubations, laccase activity increased with time (day 2, 0.2 U/liter; day 12, 97 U/liter). C. vulgaris produced less than 2 U/liter of laccase (Fig. 6B). Single cultures of T. versicolor 159, T. pubescens 220 and S. vacuolatus did not appear to produce any laccase under these conditions.

### Glucose-6-phosphate concentrations.

**FIG 7:**
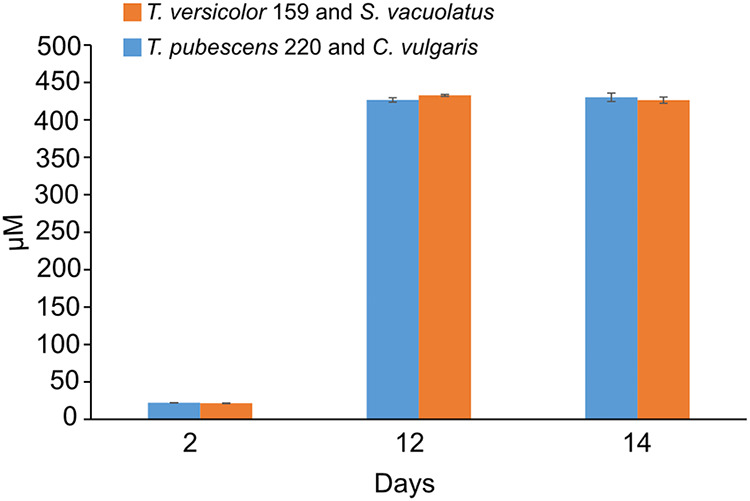
Glucose-6-phosphate concentrations at different days of the incubation period of cocultures when incubated with 1:1 SK:ME medium mixture and TEMPO-oxidized CNF (0.017% wt) and CNC (0.2% wt) under ambient conditions and red and blue artificial light. Cultures were initially shaken to stimulate growth but after 2 days they were left to stand for the duration of the experiment. Data represent an average of three biological replicates.

To assess the metabolic activity of cocultures in the presence of cellulose we measured glucose-6-phosphate (G-6-P) concentrations. G-6-P was produced as part of glycolysis, the pathway that breaks down glucose. Concentrations of G-6-P increased in cocultures between the first and last 2 days of the experiment (Fig. 7). G-6-P concentrations were similar between T. versicolor 159 and S. vacuolatus cocultures (day 2, 22 μM; day 14, 430 μM) and T. pubescens 220 and C. vulgaris cocultures (day 2, 22 μM; day 14, 426 μM).

## DISCUSSION

Our combined microscopy results failed to show evidence of internalization of algae cells into the filaments of white-rot fungi. Instead, we observed that algae appear to bind to the filaments of white-rot fungi (Fig. 1), they are embedded in a biofilm matrix containing fungal filaments (Fig. S1 and S2A in Supplemental File 1) or are simply present in the sample with the fungus (Fig. 3). Whether the interaction between the two is mutualistic or antagonistic is not clear. The results from our growth experiments (Fig. 4A to C and E, Fig. S1 and S3 in Supplemental File 1) in the presence of cellulose demonstrate that nanocellulose in the form of CNC and CNF does not appear to be toxic to the algae and fungi. These results are in line with another study showing that TEMPO-oxidized CNF hydrogels are compatible with green algae and with our previous results with the fungi alone (15, 17). A comparison of 3D-printed inks with and without cells indicated that the cell structures surrounding the mycelia or attached to the mycelia were algae (Fig. 4A to F). In this hydrogel system, fungal growth appeared to outcompete algal growth. Nevertheless, the results showed that single cultures of fungi or fungal-algal cocultures grew at comparable growth rates in the TEMPO-CNF and CNC hydrogels (Fig. 4G). Thus, this material is biocompatible with these cocultures. Future experiments could explore the effect of increasing the algal cell load in the cellulose mixture and whether this would shift the growth in favor of both organisms and promote closer interaction between fungi and algae.

The growth experiments on agar plates show that in the absence of any nutrient source, the growth of the fungal mycelium in both cocultures is slower than when the cocultures are grown under nutrient-rich conditions (Fig. 2A and B). In particular, the controls containing only fungi, grow faster without any nutrients in the agar incubations (Fig. 2B). One possible explanation why cocultures grow slower in the absence of nutrients could be that nutrient depletion triggers the fungus to feed on the algae or feed off sugars or nutrients produced by the algae at the center of inoculation. Thus, the fungus spends more time feeding at the center of inoculation before growing outwards. However, in the presence of nutrients, the fungus may co-exist with the algae, allowing it to grow at normal rates but not necessarily benefit from this interaction. In a cocultivation study (9), SEM images (see Fig. 1E and F in reference [9]) show fibrous extensions emanating from the algae N. oceanica while attached to the hyphae of the fungus M. elongate, an indication that it is missing a cell wall. The authors speculated that it was the fungus-produced enzymes that dissolved away the cell wall of the algae, thereby facilitating its internalization. Our SEM images of T. versicolor 159 and S. vacuolatus cells grown in 1:1 SK:ME media, showed cells of S. vacuolatus that were still intact while attached to filaments of T. versicolor 159 (Fig. S2A and B in Supplemental File 1). This would imply, that at least for this pair, the interaction does not trigger the dissolution of S. vacuolatus by T. versicolor 159.

The increased production of endoglucanase and laccase in cocultures of T. versicolor 159 and S. vacuolatus (Fig. 5 and 6; Table 1) indicated that the presence of the algae acted as a stimulant. Previous studies have shown that when fungi are cocultivated with other fungal partners or bacteria, laccase production can be enhanced possibly as a defense mechanism (28–31). These interactions can lead to increased expression of silent genes and diverse secondary metabolites in cocultures of fungi with other microbes (32). However, not all interactions result in increased production of lignocellulose enzymes (30, 33, 34). This seems to be the case with the cocultures of T. pubescens 220 and C. vulgaris. While endoglucanase production was high in these cocultures, laccase production diminished over time (Fig. 5 and 6, Table 1). Whether living algal cells are needed to stimulate enzyme production by white-rot fungi is not clear based on our results. Therefore, future studies should address whether algal cell wall components or dead algal cells could also trigger enzyme production by their cocultivation partner. The low level of laccase activity observed in the algae incubations may be attributed to laccase or laccase-like enzymes. A few studies have observed laccase-like phenoloxidase activity by algae inhabiting soils and by S. vacuolatus during biofuel production (35, 36). It is also possible that T. versicolor 159 stimulates S. vacuolatus to produce laccase and, hence, the increase of laccase activity over time.

**TABLE 1:** Enzyme activities in units (U) and concentrations in cocultures and fungi

Organism	Growth conditions	Endoglucanase activity	G-6-P activity	Laccase activity	Reference
T. versicolor 159	TEMPO-oxidized CNF (0.017% wt) and CNC (0.2% wt) (2% [wt/vol] ME, 25°C, 80% RH)^*a*^	~15 ± 1 U/mL (5 days) ~6 ± 2 U/mL (14 days)	<40 μM	~13 ± 7 U/liter (5 days) ~339 ± 42 U/liter (14 days)	15
T. versicolor 159	TEMPO-oxidized CNF (0.017% wt) and CNC (0.2% wt) (1:1, SK:ME), RT, LD cycles	~1 ± 0 U/mL (2 days) ~3 ± 0 U/mL (7 days)	NM	BDL	This study
T. versicolor 159 and S. vacuolatus	TEMPO-oxidized CNF (0.017% wt) and CNC (0.2% wt) (1:1, SK:ME), RT, LD cycles	~37 ± 2 U/mL (2 days) ~5 ± 1 U/mL (12 days)	426 ± 4 μM	2 ± 0 U/liter (2 days) 99 ± 12 (12 days)	This study
T. pubescens 220	TEMPO-oxidized CNF (0.017% wt) and CNC (0.2% wt) (2% [wt/vol] ME, 25°C, 80% RH)	~14 ± 0 U/mL (5 days) ~8 ± 4 U/mL (14 days)	<40 μM	~130 U/liter ± 15 (5 days) ~37 U/liter ± 24 (14 days)	15
T. pubescens *220*	TEMPO-oxidized CNF (0.017% wt) and CNC (0.2% wt) (1:1, SK:ME) RT, LD cycles	~1 ± 0 U/mL (2 days) ~4 ± 0 U/mL (12 days)	NM	BDL	This study
T. pubsescens 220 and C. vulgaris	TEMPO-oxidized CNF (0.017% wt) and CNC (0.2% wt) (1:1, SK:ME) RT, LD cycles	~36 ± 2 U/mL (2 days) ~3 ± 0 U/mL (12 days)	430 ± 6 μM	10 ± 5 U/liter (2 days) 0.2 ± 0 U/liter (12 days)	This study

a NM, not measured; LD, light and dark cycles; RH, relative humidity; RT, room temperature; BDL, below the detection limit.

The increase in G-6-P concentrations in both cocultures over time indicates an increase in glucose breakdown via the glycolysis pathway (37) (Fig. 7 and Table 1). Glucose could be produced from the breakdown of cellulose by the white-rot fungus, as a result of glucose production from photosynthesis by the algal partner or it could be present in the malt extract (15, 38). When cellulose is metabolized by white-rot fungi, it is first broken down into cellobiose by 1,4-β-d-cellobiohydrolase, and by 1,4-β-d-endoglucanases. Cellobiose, in turn, is broken down into glucose or glucose oligosaccharides by beta-glucosidase (39). These types of enzymes can be secreted by white-rot fungi (40). Something to note is that high concentrations of monomeric sugars such as d-glucose and d-fructose have been shown to inhibit the production of cellulase in white-rot fungi (41, 42). However, the glucose concentration needed to repress cellulase genes in individual species and strains is a question that needs to be addressed in future studies, not only with the white-rot fungi studied here but in general (42). While glucose can repress cellulase activity, other factors such as nitrogen concentration, light, and cellulose can stimulate the expression of cellulase enzymes (15, 43, 44). The results here suggested that the factors that stimulate cellulase activity, specifically with respect to endoglucanases, may counteract the mechanisms involved in these fungi that would normally repress their activity in the presence of glucose.

Overall, our study demonstrated that cocultures of T. versicolor 159 and S. vacuolatus and cocultures of T. pubescens 220 and C. vulgaris could grow in the presence of TEMPO-oxidized CNF and CNC in liquid and hydrogel systems. Thus, TEMPO-oxidized CNF and CNC were biocompatible with the cocultures. When cultivated under static conditions in 1:1 SK:ME in the presence of nanocellulose, the cocultures produced more lignocellulose enzymes compared to single cultures of white-rot fungi. Additionally, T. versicolor 159 and S. vacuolatus produced more endoglucanases over time under these conditions. The results of this study showing enhanced lignocellulose production by these cocultures could pave the way for their use in the degradation of lignocellulose materials. This application would especially be beneficial to industries that produce large amounts of lignocellulose waste, including the food (45) and paper industries (46). Future studies aimed at studying the 3D-living architecture of embedded cocultures can consider using our cellulose hydrogel system as an alternative option rather than a synthetic hydrogel system. Furthermore, our 3D-printed hydrogel system offers an alternative option rather than suspension cell culture methods to study fungal-algal consortia.

## MATERIALS AND METHODS

### Microorganisms and their growth conditions.

The freshwater algae C. reinhardtti 21 gr(+) (47), Chlorella vulgaris 9–88 (UAM 101) (48), and S. vacuolatus SAG211 to 15 (SAG culture collection https://uni-goettingen.de/en/www.uni-goettingen.de/de/184982.html) were grown in a modified version of the Sueoka (SK) medium (49). One liter of medium contained KH_2_PO_4_ (8.3 mM), K_2_HPO_4_ (5.3 mM), MgSO_4_ 7H_2_O (0.25 mM), CaCl_2_ H_2_O, (88 μM), 5 mL of Hutner’s trace metal solution (50), and 1.5 g of KNO_3_. Algae were grown at ambient light and room temperature (RT). The white-rot fungi Trametes versicolor (Empa strain 159), Trametes pubescens (Empa strain 220), Ganoderma adspersum (Empa strain 003), Ganoderma lipsiense (Empa strain 646), and Rigidoporus vitreus (Empa strain 643) were grown in 2% malt extract (ME) medium at 80% relative humidity (RH) and 27°C. Taxonomic names of the fungi are based on the latest entries in Mycobank (http://www.mycobank.org, accessed on March 27, 2022) and taxonomic names of the algae are based on the latest entries in Algae Base (https://www.algaebase.org, accessed on October 27, 2021).

### Growth of algae and white-rot fungal algae cocultures.

Cocultures were prepared by mixing 250 μL of the various freshwater algae and white-rot fungi from above in sterile Eppendorf tubes and letting them incubate at room temperature (RT) under ambient light conditions for 6 months. After 6 months, the contents of the Eppendorf tubes were transferred to 50 mL sterile falcon tubes containing a 1:1 SK:ME medium mixture. Medium mixtures have been used for the cocultivation of various species of bacteria, fungi, and algae (23). The medium mix and tubes were agitated at 120 rpm under ambient light conditions and at RT for several days. Cocultures were continuously subcultured in a 1:1 SK:ME medium mixture using the conditions described above. When used as controls, the white-rot fungi were also cultivated in the 1:1 SK:ME medium mixture under the same conditions.

### Light microscopy images of cocultures.

Following 21 days of cultivation, cocultures were sampled by taking a lancet and breaking off pieces of biomass growing in the Eppendorf tubes, and placing this sample on a microscope slide with a cover slip. Microscope images were made using a Leica microscope (model DSM4000B-M) with a Leica HC PL FLUOTAR 10×/0.30 and 20×/0.50 DRY lens objectives. The following microscope parameters were used: TL-PH Σ100x INT 15 AP 38 FD 33. Images were captured with the LAS AF Software 3.6.0.20104. Scale bars were added to images postprocessing using the open-source software Fiji (51) and ImageJ (52).

### Transmission electron microscopy images of cocultures.

For TEM imaging, samples were first dissected with forceps and a scalpel using a dissecting microscope. Cross-sections were extracted from the central region of the fungal pellets. Next, samples were placed in plastic wells resistant to acetone and fixed with 2.5% glutaraldehyde and 2% formaldehyde in 0.15 M Na-cacodylate buffer, supplemented with 2 mM calcium chloride. The samples were then washed three times in Na-cacodylate buffer and postfixed with 1% osmium tetroxide and 1% aqueous uranyl acetate. Next, samples were dehydrated in steps using 50, 2 × 75, 90, 98, and 3 × 100% acetone (dried over a molecular sieve), before embedding in Epon (Fluka Epoxy Embedding kit). For this, samples were infiltrated twice with 25% Epon in dry acetone, followed by two changes, each of 50% and 75% Epon, fixation, and all following steps until resin infiltration with 75% Epon was performed in a microwave sample processor (Pelco BioWave, Ted Pella Inc., CA, USA). Then samples were left in 100% Epon overnight in the fridge, then transferred twice into fresh Epon for approximately 1 h. Samples were cut into smaller pieces (<1 mm in diameter) before placing them into molds with fresh Epon and polymerization at 60°C for 3 to 4 days.

Thin sections of 50 nm were obtained with a diamond knife (Diatome Ltd., Switzerland) on a Leica UC7 ultramicrotome (Leica Microsystems, Heerbrugg, Switzerland), placed on Formvar and carbon-coated TEM grids (Quantifoil, Groβlöbichau, Germany), and poststained with 2% uranyl acetate and Reynold’s lead citrate for 30 s each. Stained sections were then visualized using a Morgagni 268 TEM at 100 kV (Thermo Fisher Scientific, Waltham, MA, USA).

### Cellulose ink preparation.

To monitor the growth of cocultures on the surface of printed inks using bright microscopy (described below), an ink containing TEMPO-oxidized CNF (1.2% wt) and sulfuric acid hydrolyzed CNC (10% wt) was prepared by combining 0.48 g of TEMPO-oxidized CNF, 4 g of CNC, and 34 g of water with a resistivity ≥ 18 MΩ cm^−2^ in a 125 mL SpeedMixer container. The ink was first mixed with a spatula and then using an orbital mixer (Hauschild and Co. KG, SpeedMixer DAC 600) at 1500 rpm for 1 min and 2300 rpm for 4 min before storing it overnight at 4°C to allow it to swell. 0.8 g of malt extract (Oxoid) was added to the ink, mixed with a spatula, and orbital mixed at 2300 rpm for 1 min. The ink was autoclaved for 15 min at 100°C (Systec VX-121). Inks were stored at 4°C until ready for printing.

### Cellulose ink printing.

TEMPO-oxidized CNF and CNC were printed using a direct ink writing (DIW) Ultimaker 2+ instrument (my3DWorld GmbH, Switzerland) enclosed in a plexiglass enclosure to maintain sterile conditions during printing steps. The cellulose ink was loaded into a sterile 20 mL plastic Luer-lock syringe inside a laminar flow-hood using a sterile spatula. The syringe was capped with an ethanol sterilized syringe barrel end cap (S&P Shop, H. Sigrist & Partner AG, Switzerland) and then centrifuged for 5 to 10 min at 3,000 rpm (Rotina 380, Hettich AG, Switzerland) before printing. At the printing step, the end cap was replaced with an ethanol-sterilized, 0.84 mm diameter, tapered dispensing tip (S&P Shop, H. Sigrist & Partner AG, Switzerland). Printing was carried out at room temperature. The printing platform and the plexiglass enclosure were sterilized with 70% ethanol before printing.

### Growth of cocultures on cellulose printed inks.

Biomass of C. vulgaris and T. pubescens 220 and biomass of S. vacuolatus and T. versicolor 159 were cut into pieces and weighed. These pieces were used to inoculate the center of 10% TEMPO oxidized CNF and CNC printed discs (5 cm diameter, ~1 mm thick) with malt extract. The inks were prepared as described above. Cultures were grown at room temperature with ambient light. The growth rate of the coculture biomass on top of the hydrogel was tracked by measuring the fungal mycelial growth outward with time. The growth of the coculture biomass was also determined using bright field microscopy.

### Enzyme assays.

Cultures were grown in 70 mL of SK:ME 1:1 medium mixture supplemented with 1g TEMPO-oxidized CNF (1.2% wt) and sulfuric acid hydrolyzed CNC (15% wt) giving a final concentration of CNF and CNC 0.017% wt and 0.2% wt, respectively. TEMPO-oxidized CNF and CNC were prepared as described previously (53, 54) and added to commercially purchased CNC (Celluforce, Canada). Cultures were initially shaken for 2 days at 120 rpm to promote the growth of the coculture and then left to stand at ambient RT. Cocultures were grown under an LED light (as less light was available for growth during the fall) (Hama Stick; blue-red setting, 9 h cycle). At different time intervals, 1.5 mL of supernatant was removed and the tubes were frozen until further analysis. Before analysis, biomass was removed by centrifuging thawed tubes at 10,000 rpm for 10 min. The supernatants were transferred to new test tubes and endoglucanase, laccase, and glucose-6-phosphate assays were performed as previously described (15).
