# Supplementary material for: Cocultivation of White-Rot Fungi and Microalgae in the Presence of Nanocellulose
Source: Microbiol Spectr. 2022 Sep 26;10(5):e03041-22. doi: 10.1128/spectrum.03041-22 (PMC9604150; doi:10.1128/spectrum.03041-22)
Supplement: Supplemental file 1 — Supplemental material. Download spectrum.03041-22-s0001.pdf, PDF file, 0.7 MB [file spectrum.03041-22-s0001.pdf]

Supporting Material: Co-cultivation of white-rot fungi and microalgae in the presence of nanocellulose

[a]Carolina Reyes, [a]Zsófia Sajó, [b]Miriam Susanna Lucas, [ad]Ashutosh Sinha, [c]Francis W.M.R. Schwarze, [c]Javier Ribera, [ad]Gustav Nyström

[a]Laboratory for Cellulose and Wood Materials, Empa, Überlandstrasse 129, 8600 Dübendorf, Switzerland

[b]Scientific Center for Light and Electron Microscopy (ScopeM), ETH Zurich, Otto-Stern-Weg 3, HPM C57.2, 8093 Zurich, Switzerland

[c]Laboratory for Cellulose and Wood Materials, Empa, Lerchenfeldstrasse 5, 9014 St. Gallen, Switzerland

[d]Department of Health Science and Technolgy, ETH Zürich, Schmelzbergstrasse 9, 8092 Zürich, Switzerland

Correspondence: creyes6@gmail.com (C.R), gustav.nystroem@empa.ch (G.N.)

Supporting Methods

Sample preparation and SEM imaging of *T. versicolor* 159 and *S. vacuolatus*

T. versicolor and *S. vacuolatus* were inoculated in 70 mL of 1:1 media mixture supplemented with 1g TEMPO-oxidized CNF (1.2 wt%) and sulfuric acid hydrolyzed CNC (15 wt%) giving a final concentration of CNF and CNC 0.017 wt% and 0.2 wt% respectively. TEMPO-oxidized CNF and CNC was prepared as described previously (1)(2) and added to commercially purchased cellulose nanocrystals (CNC) (Celluforce, Canada). Cultures were initially shaken for 2 days at 120 rpm to promote the growth of the co-culture and then left to stand at ambient room temperature. Co-cultures were grown under an LED light (as less light was available for growth during the fall) (Hama Stick; blue-red setting, 9 hour cycle). Over time, the co-culture formed a mat-like structure at the surface of the liquid medium. This structure was carefully transferred to a petri dish, frozen by the addition of liquid nitrogen, and lyophilised overnight (Alpha 3-4 LSCbasic). A small portion of the lyophilised sample (approximately 1 cm x 2 cm) was cut, coated with a 7nm layer of platinum (Bal-Tec Med 020), and assessed via SEM (FEI QUANTA 650FEG ESEM).

Growth of co-cultures inside printed inks

To monitor the growth of co-cultures inside printed inks using confocal microscopy, TEMPO-oxidized CNF (1.2 wt%) and sulfuric acid hydrolyzed CNC (10 wt%)) were prepared as described above, except that after the addition of the malt extract, the ink was orbital mixed at 1500 rpm for 1 min and 2300 rpm for 4 min and stored at 4°C in the dark until cell transfer. Sulfuric acid hydrolyzed CNC (20 wt%) was prepared using 1:1:SK:ME by combining 16.44 g CNC and 80 mL of 1:1:SK:ME, mixed with a spatula and orbital mixed at 1500 rpm 1 min and 2300 rpm for 4 min. The ink was

stored for 2-3 days at 4°C. Next, 40 g of the ink was orbital mixed for 1 min before autoclaving as described above. During cell transfer, 10^5 *C. vulgaris* cells, grown in Sueoka`s high salt medium were transferred to 40 g of ink by diluting cells in 1X PBS solution to a final volume of 100 µL. The ink was mixed by orbital mixing the ink at 1500 rpm for 1 min and 2300 rpm at 4 min. Algae cell counts were determined using a Neubauer chamber and a Zeiss Axioplan optical microscope with trans-illumination and an Epiplan NEOFLUR 10X objective. *T. pubescens* 220 was transferred to inks by scraping off 0.02 g of mycelium from a 2% MEA plate. The sample was transferred to a 50 mL sterile Falcon tube and 5 mL of 1X PBS added along with sterile glass beads (Sigma, 2 mm diameter). The suspension was mixed (Vortex Genie 2, Scientific Industries, 2 min, level 10) until the fibers broke apart. 2 mL of this suspension was added to the ink and orbital mixed as above before storing the ink at 4°C in the dark.

Confocal laser scanning microscopy (CLSM) images of printed inks

TEMPO-oxidized CNF and CNC printed inks containing co-cultures were prepared by using a 5 nM SYTOX Green solution, prepared in Hank`s Balanced Salt Solution (HBSS) buffer according to the manufacturer`s instructions and following previous studies (3)(4). Gels were placed in glass bottom dishes and washed once with 1 mL HBSS. Next, they were incubated in the dark for 15 minutes in 5 nM SYTOX Green solution, washed three times using 1 mL HBSS and stored at room temperature until imaging.

The dye had an excitation wavelength of 504 nm and an emission wavelength of 523 nm. Samples were imaged using a LSM 780 confocal laser scanning microscope (Carl Zeiss, Oberkochen, Germany). Images were acquired with the ZEN 2012

software (Carl Zeiss). During imaging, an Argon and HeNe633 laser were used. The beam filter MBS 488/561/633 was used for imaging. AlexaFluor 488 detection wavelengths were 493-606 nm (detecting SYTOX Green emission) and AlexaFluor 633 detection wavelengths were 647-721 nm (detecting Chlorophyll A emission). Plan Aprochromat 10x.0.45 M27 objective or LD Plan NeoFluor 20x/0.4 Korr objectives were used for imaging. Bright field microscopy was used alongside fluorescence microscopy imaging.

Confocal laser scanning microscopy (CLSM) images of co-cultures

For confocal microscopy fungal pellets with attached algae that were growing for 23 days in the 1:1 ME:SK media mixture, were cut into pieces with a sterile scalpel. These pieces were centrifuged in a 1.5 mL Eppendorf tube at 10,000 rpm (Eppendorf MiniSpin Benchtop Centrifuge) for 10 min to remove the media before proceeding to the washing step. For the washing step sterile Hanks` Balanced Salt Solution (HBSS) buffer was used according to the manufacturer`s instructions. 1mL of HBSS was added to the biomass in the Eppendorf tube and the sample centrifuged as above. This was repeated a second time. After, 2 mL of 10 nM Sytox Green in DMSO (Thermo Fisher Scientific) was added to the sample in a confocal petri dish and the sample incubated for 15 minutes in the dark. Next, the dye was removed and the biomass washed once more with 2 mL of HBSS. The HBSS solution was removed prior to imaging.

The dye had an excitation wavelength of 504 nm and an emission wavelength of 523 nm. Samples were imaged using a LSM 780 confocal laser scanning microscope (Carl Zeiss, Oberkochen, Germany). Images were acquired with the ZEN 2012 software (Carl Zeiss). During imaging, Argon and HeNe633 lasers were used. The

beam filter MBS 488/561/633 was used for imaging. AlexaFluor 488 detection wavelengths were 493-606 nm (detecting SYTOX Green emission) and AlexaFluor 633 detection wavelengths were 647-721 nm (detecting Chlorophyll A emission). Plan Aprochromat 10x.0.45 M27 objective or LD Plan NeoFluor 20x/0.4 Korr objectives were used for imaging. Bright field microscopy was used alongside fluorescence microscopy imaging.

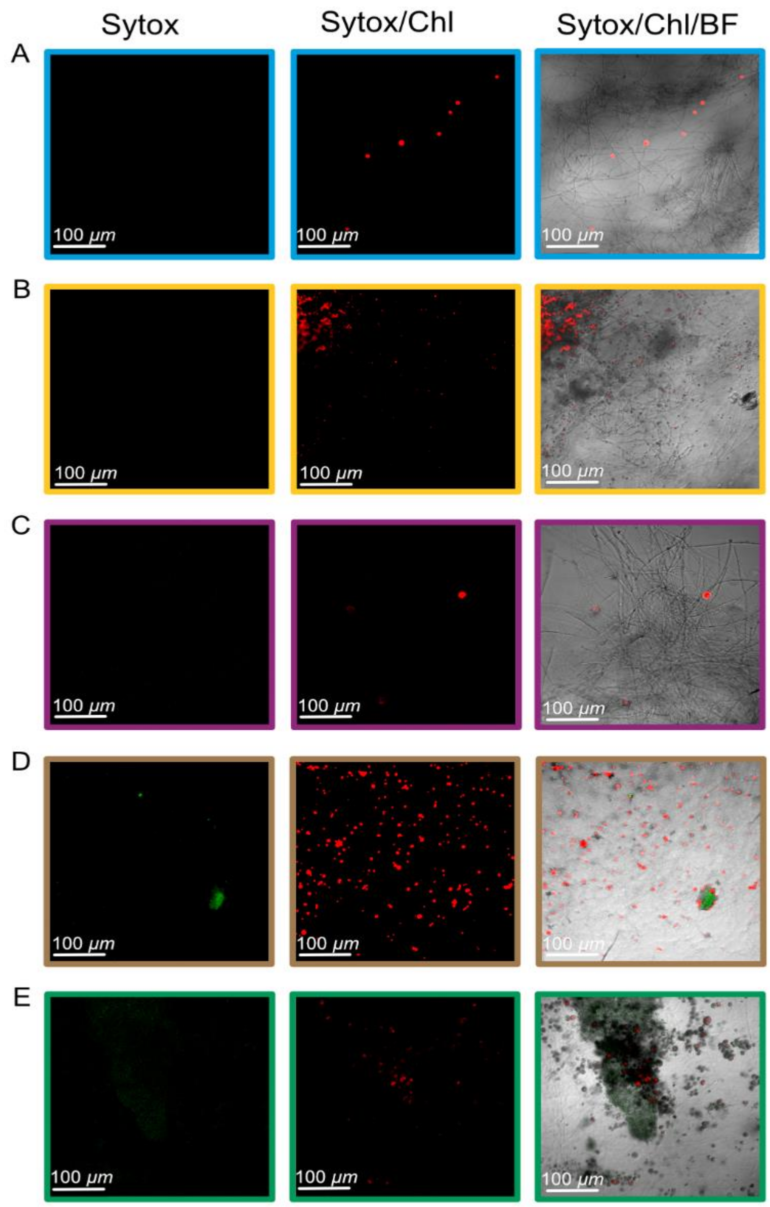

Figure S1. Laser scanning confocal microscopy images of fungi and algae co-cultures after ~ 23 days of growth in 1:1 SK:ME media. Dead fungal cells are indicated by SYTOX Green staining (green fluorescence). Red cells are chlorophyll pigments of algae cells that autofluoresce in living and instantly killed cells but not in disintegrating dead cells (5). (A, blue) T. pubescens 220 and C. vulgaris, (B, yellow) R. vitreus 643 and C. vulgaris, (C, purple) T. versicolor 159 and S. vacuolatus, (D, brown) G. adspersum 003 and S. vacuolatus, (E, green) R. vitreus 643 and S. vacuolatus. The beam filter MBS 488/561/633 was used for imaging. All rows, except the fourth row, were imaged using LD Plan NeoFluor 20x/0.4 Korr objective. The fourth row was imaged using Plan Aprochromat 10x.0.45 M27 objective.

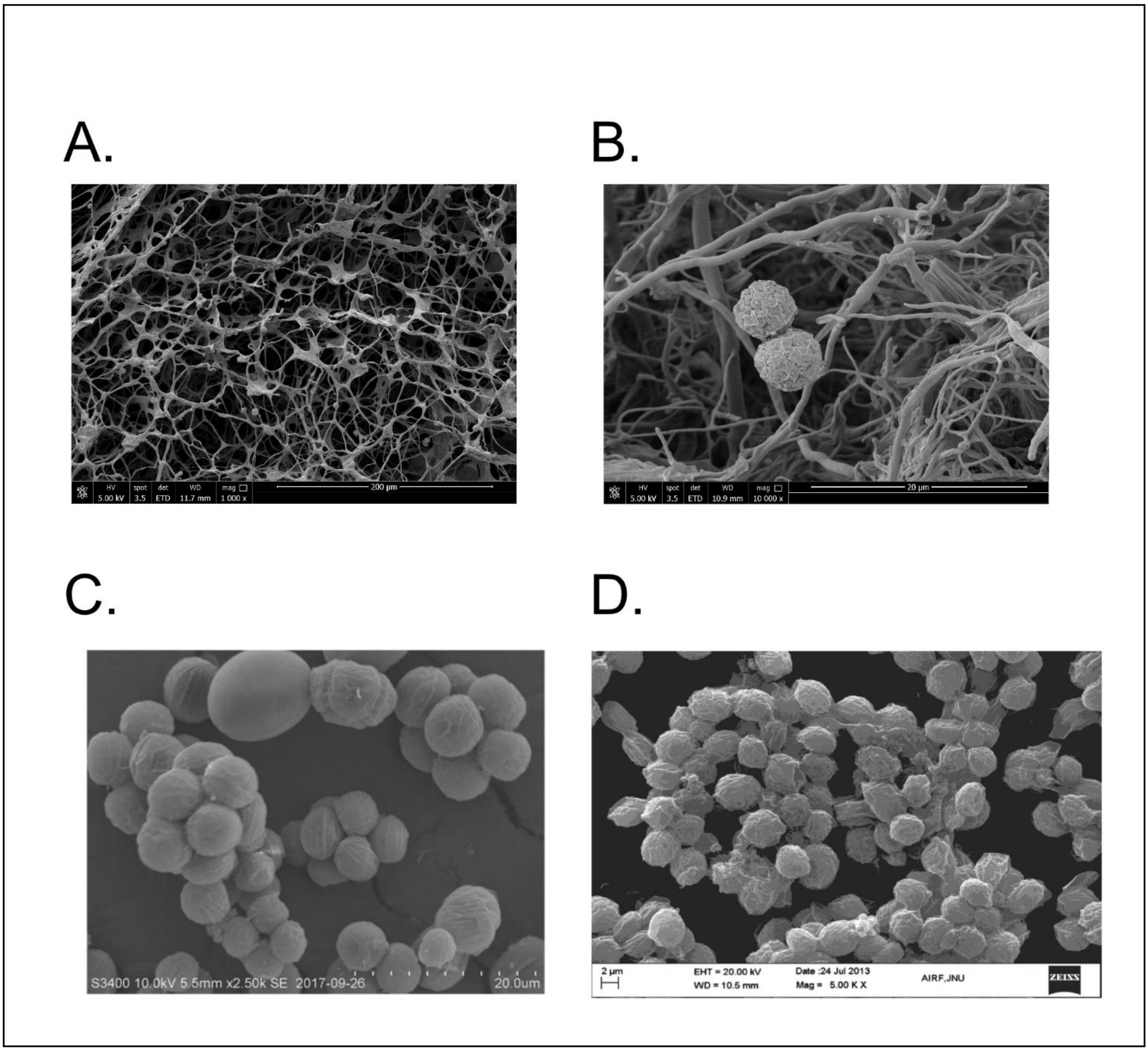

Figure S2. Scanning electron microscopy images. A) Overview of the filamentous structure with round *S. vacuolatus* cells distributed in *T. versicolor* filaments. B) Close-up of *S. vacuolatus* cells ontop of a fungal *T. versicolor* filament. (C) SEM image of *Scenedesmus obliquus* AS-6-11 for morphology comparison to *S. vacuolatus* cells (6) (figure reused from Chen et al. (2020) BMC Genomics with open access permission from the publisher). (D) SEM image of *Scenedesmus* sp. ISTGA1 for morphology comparison to *S. vacuolatus* (7) (figure reused from Tripathi et al. (2014) Renewable Energy with permission from the publisher).

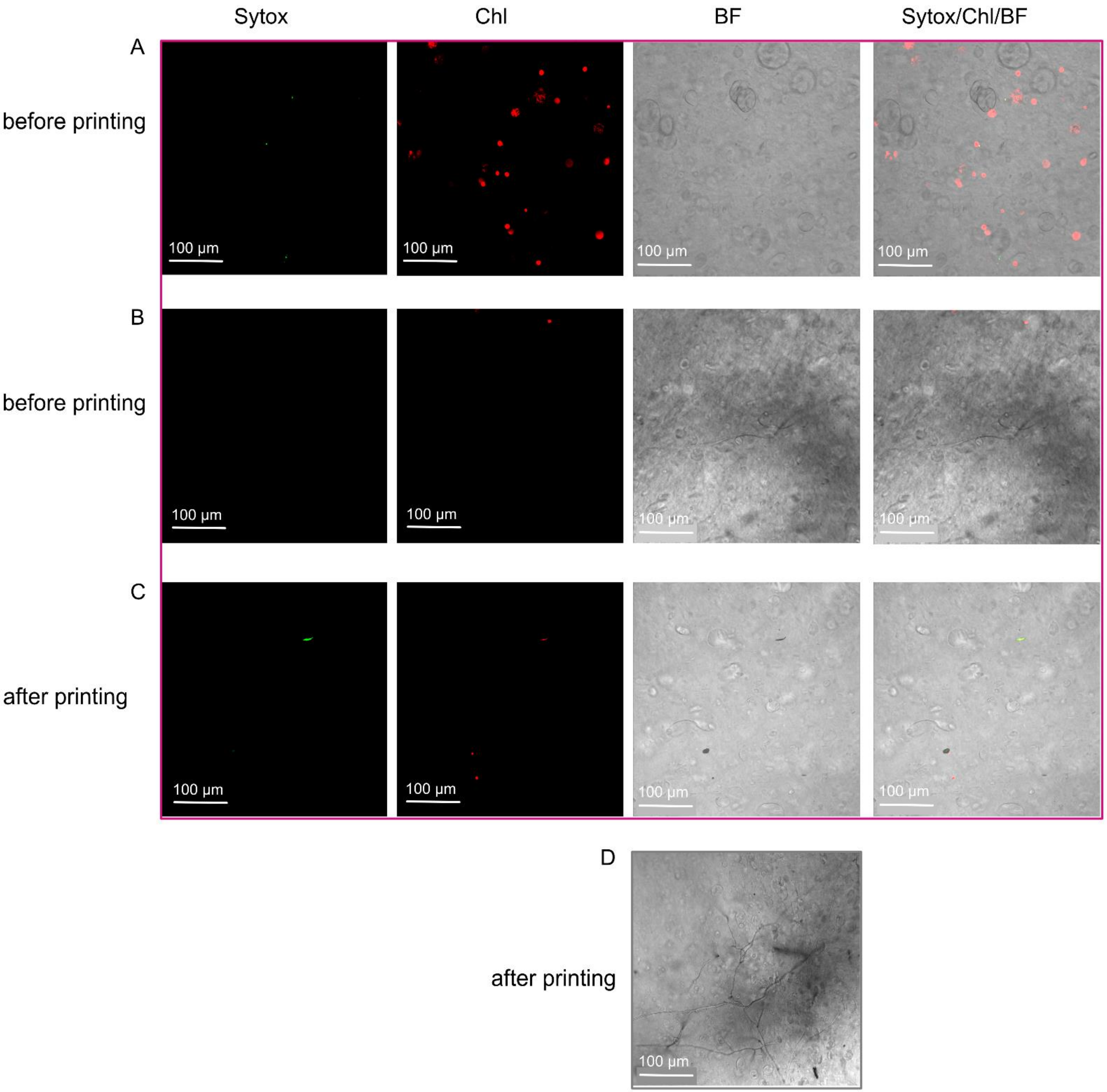

Figure S3. Confocal microscopy images of (A-C) *T. pubescens* 220 and *C. vulgaris* or (D) *T. pubescens* after mixing into 20 wt% (LD Plan NeoFluor 20x/0.4 Korr objective) hydrogel before and after printing steps. Dead fungal cells are indicated by SYTOX Green staining (green fluorescence). Red cells are chlorophyll pigments of algae cells that autofluoresce in living and instantly killed cells but not in disintegrating dead cells (5). BF indicates bright field, Chl indicates chlorophyll A.

References

1. Saito T, Kimura S, Nishiyama Y, Isogai A. 2007. Cellulose nanofibers prepared by TEMPO-mediated oxidation of native cellulose. Biomacromolecules 8:2485–2491.
2. Weishaupt R, Siqueira G, Schubert M, Tingaut P, Maniura-Weber K, Zimmermann T, Thöny-Meyer L, Faccio G, Ihssen J. 2015. TEMPO-oxidized nanofibrillated cellulose as a high density carrier for bioactive molecules. Biomacromolecules 16:3640–3650.
3. Du ZY, Zienkiewicz K, Pol N Vande, Ostrom NE, Benning C, Bonito GM. 2019. Algal-fungal symbiosis leads to photosynthetic mycelium. Elife 8:10–12.
4. Johnston TG, Fillman JP, Priks H, Butelmann T, Tamm T, Kumar R, Lahtvee PJ, Nelson A. 2020. Cell-laden hydrogels for multikingdom 3D printing. Macromol Biosci 20:1–7.
5. Du ZY, Zienkiewicz K, Pol N Vande, Ostrom NE, Benning C, Bonito GM. 2019. Algal-fungal symbiosis leads to photosynthetic mycelium. Elife 8:1–22.
6. Chen BL, Mhuantong W, Ho SH, Chang JS, Zhao XQ, Bai FW. 2020. Genome sequencing, assembly, and annotation of the self-flocculating microalga *Scenedesmus obliquus* AS-6-11. BMC Genomics 21:1–14.
7. Tripathi R, Singh J, Thakur IS. 2015. Characterization of microalga *Scenedesmus* sp. ISTGA1 for potential CO2 sequestration and biodiesel production. Renew Energy 74:774–781.
